# Supplementary material for: International corneal and ocular surface disease dataset for electronic health records
Source: Br J Ophthalmol. 2025 Jun 15;109(10):e327110. doi: 10.1136/bjo-2024-327110 (PMC12408911; doi:10.1136/bjo-2024-327110)
Supplement: online supplemental file 1 [file bjo-109-10-s001.docx]

**Supplementary Table 1.** Summary of countries of the international experts involved.

| Countries |
| --- |
| Australia  Austria  Canada  Denmark  France  Germany  India  Italy  Japan  New Zealand  Pakistan  Russia  Singapore  Spain  United Kingdom  United States of America |
